# Genotoxicological Evaluation of NUTRALYS Pea Protein Isolate

**DOI:** 10.1155/2013/817353

**Published:** 2013-02-27

**Authors:** Chentouf Aouatif, Ph. Looten, M. V. S. Parvathi, S. Raja Ganesh, V. Paranthaman

**Affiliations:** ^1^Biology and Nutrition Department, Roquette Frères, 62136 Lestrem, France; ^2^International Institute of Biotechnology and Toxicology (IIBAT), Padappai, Tamil Nadu 601301, India

## Abstract

NUTRALYS Pea Protein Isolate, a protein supplement, is a high-quality source of protein which is primarily emulsifying functional protein. We evaluated the genotoxic potential of NUTRALYS isolated from dry yellow pea, using three established genotoxicity tests (AMES test *in vitro* chromosomal aberration test, and *in vivo* micronucleus test) employing OECD guidelines under GLP conditions. In the bacterial reverse mutation test, NUTRALYS did not show positive responses in strains detecting point and frame shift mutations. In the chromosomal aberration test, NUTRALYS did not induce chromosome aberrations in the presence and absence of metabolic activation. In the bone marrow micronucleus test, NUTRALYS did not induce significant increases of micronucleated immature (polychromatic) erythrocytes in bone marrow of test animals.

## 1. Introduction

Human food contains essential nutrients that make up the source of metabolism and energy. For improvement of the health, additional ingredients are consumed in day-to-day life. For this purpose, many formulations are produced, and several natural sources are added to nutritional supplements in different forms to compensate the shortage of some nutrients. The use of nutritional supplements has remarkably increased in recent years [1].

NUTRALYS Pea Protein Isolate (Food grade supplement—by Roquette Frères) is a high-quality source of protein is extracted from the dry yellow pea which is primarily emulsifying functional protein. It is a vegetable protein rich in amino acids used in high protein foods and drinks. The pea is subjected to a wet process treatment. This process ensures that pea isolates with 85% to 90% protein are obtained, with a high level of functionality. It is a creamy colour powder, with protein content of 85%.

To ensure the safe use and quality of dietary supplements, the FDA [2] recently announced regulations for good manufacturing practices for nutritional supplements. Recent studies projected plants to be important sources of biologically active products, many of which serve as models for the synthesis of a large number of medicines. Hence scientific studies have enabled use of plant products in the therapeutics of different diseases [3]. Usually consumers of food products do not find time or capacity to assess biochemical and safety data on every substance they consume or to assess for themselves on a daily basis the chemical components of every product they are supposed to be purchasing. In the case of food additives, Congress has entrusted the Food and Drug Administration (FDA) to direct the safety of food ingredients, including the premarket safety evaluation of new food additives intended for our foods. In this way consumers are unchained to make their own verdict on a product-by-product basis for the food ingredient safety issues each time they make a purchase [4].

According to Food Safety Commission of Japan (FSCJ), 2010 [5], substances that are to be used as an alternative to ordinary food ingredients or for the purpose of nutritional enhancement or as a “food with nutritional claims” must be examined regarding their quality as a nutritional component as well as relatively to the intake levels of the same nutritional components that are available in other foods. It is preferable to examine as necessary *in vitro *studies and other studies conducted during pharmaceutical development or in other areas that are recommended for food additive studies. Genetic toxicity tests included in the standard combination are bacterial reverse mutation tests, chromosome aberration tests using cultured cells of mammals, and micronucleus tests on rodents. In all the three tests, NUTRALYS is considered to be nongenotoxic and safe to be used as dietary supplement.

## 2. Material and Methods

NUTRALYS Pea Protein Isolate (Food grade supplement—by Roquette Frères) is a high-quality source of protein extracted from the dry yellow pea which is primarily emulsifying functional protein. It contains 85% to 90% protein with a high level of functionality. It is a creamy colour powder, with protein content of 85%.

Genetic toxicity tests included in the standard combination are bacterial reverse mutation tests, chromosome aberration tests using cultured cells of mammals, and micronucleus tests on rodents. All the tests were conducted at IIBAT, Padappai, India, a GLP compliant laboratory following suitable OECD guidelines after prior approval from Institutional Ethical Committee.

### 2.1. Ames Test

In order to assess the mutagenic potential study was performed with five tester strains of *Salmonella typhimurium* (TA100, TA102, TA1535, TA98, and TA1537, procured from Defence Research and Development Establishment (DRDE), Gwalior, India) tested in presence and in absence of metabolic activation. The assay was performed according to OECD Guideline 471, adopted July 21, 1997. The protein was dispersed in sterile millipore water, and the test solutions were thoroughly vortexed for homogeneity, before testing. In order to choose the range of doses for the test, the cytotoxic activity of the test item was determined. The cytotoxicity assay was carried out in strains TA98 and TA100 under the same conditions as the mutagenicity test with and without metabolic activation, using 2 plates per dose. Cytotoxicity was checked by microscopic examination of the background lawn. Seven test concentrations were prepared with 78.13, 156.25, 312.5, 625, 1250, 2500, and 5000 *μ*g/plate, separated by factor 2 spacing in the preincubation method in the presence and absence of S9. The plates were incubated for approximately 48–72 hours at 37°C, and the revertants were counted using digital colony counter (Scheutt Biotec De, UK). Five test concentrations of 312.5, 625, 1250, 2500, and 5000 *μ*g/plate with 10% S9 and without S9 along with solvent and positive controls (Table 1) were chosen for mutagenicity evaluation in the five tester strains.

### 2.2. *In Vitro* Chromosomal Aberration Test

Pea Protein was evaluated for its capacity to induce structural and numerical aberrations in cultured human peripheral blood lymphocytes. The study was conducted with the approval of Institutional Ethical Committee, IIBAT. The peripheral blood was obtained from three healthy adult (>30 years age) nonsmoking male volunteers, without any recent history of illness, as is the guideline requirement. Informed consent was obtained from each donor. The assay was performed according to OECD Guideline 473, adopted July 21, 1997. The test substance was dispersed in sterile millipore water with a limit of solubility at 100 mg/mL. A range finding study was performed both in the presence and absence of S9, to choose appropriate concentrations for the main study based on solubility, precipitation, and cytotoxicity based on Mitotic Index. Seven concentrations of 15.625, 31.25, 62.5, 125, 250, 500, and 1000 *μ*g/mL with concurrent solvent controls were set. Cells were treated with the test substance for 3 hours in the presence and absence of metabolic activation system (S9), and continuously for a 36-hour period in the absence of metabolic activation system (S9), and allowed for 33-hour expression in the case of 3-hour exposure. Single cultures were treated in the range finding study. Heparinized whole blood lymphocyte cultures were established in RPMI 1640 (GIBCO) medium along with other supplements. Cultures were incubated in PHA-M (GIBCO) containing medium for 48 hours, at 37 ± 0.5°C in a humidified 5% CO_2_ atmosphere. The cultures were treated with the test substance, solvent, and positive controls in the presence and absence of S9, at 48 hours from initiation of the cultures. Cultures were incubated in a humidified 5% CO_2_ atmosphere at 37 ± 0.5°C for 1.5 cell cycle times. One hour prior to harvest, 0.4 *μ*g/mL of colchicine (Sigma, USA) was added to the cultures. The cells were pelleted, hypotonically shocked with freshly prepared and preincubated 0.075 M potassium chloride (Merck) solution at 37 ± 1°C, and washed and fixed in 3 : 1 methanol and acetic acid. Slides were prepared from cells resuspended in fixative, by dropping technique, and were air-dried. Metaphase preparations were stained with 3% Giemsa (Merck). Two hundred well-spread metaphases were scored per concentration of test substance and solvent control, equally divided amongst the duplicates using trinocular microscope (Nikon, Japan).

### 2.3. *In Vivo* Micronucleus Assay

To detect genotoxic potential of Pea Protein Isolate *in vivo,* mouse micronucleus assay was performed by assessing the induction of micronuclei in polychromatic erythrocytes (PCEs) and determining the ratio of immature and mature erythrocytes in bone marrow cells, in compliance with the OECD guideline 474, 1997. The study was conducted with the approval of Institutional Animal Ethics Committee, IIBAT. Healthy male and female CD1 mice of 6–8 weeks of age were used for the study as per guideline's specification. Solubility of the test substance was achieved in millipore water (as complete dispersion). A range finding study was performed with doses of 320, 800, and 2000 mg/kg b.w. employing 2 mice/sex/dose with concurrent vehicle control. The mice were treated orally and administered a single treatment with 24 h sacrifice time point. A limit test was performed administering single- and two-day treatments (24 h apart) with the highest dose 2000 mg/kg b.w. Mice in the positive control group were given a single treatment of 40 mg/kg b.w. cyclophosphamide monohydrate (Sigma, USA), intraperitoneally. Mice were sacrificed at 24 h and 48 h following single treatment and in case of two-day treatment; mice in the vehicle control and high dose groups were sacrificed 24 h after the final administration of test substance. Mice in the positive control group were sacrificed at the end of 24h. Both femurs from each animal were rapidly dissected out and cleaned off the adherent tissue. The epiphyses were removed to obtain access to the bone marrow canal. Bone marrow cells were flushed out with 2.5 mL FBS (Biological Industries, European grade), and the recovered cells were centrifuged at 1600 rpm for five minutes. The bulk of the supernatant fluid was discarded, and the cell pellet was resuspended in the remaining fluid with appropriate dilution. Smear was prepared by placing a single drop of the cell suspension on clean slides and then left to air-dry followed by ageing. Following fixation in methanol for ten minutes, slides were stained with (1 : 5) Giemsa solution (Merck) in water. A minimum of 2000 PCEs per mouse were scored in both sexes in each time point of sacrifice for the incidence of micronuclei. Cytotoxicity was assessed based on the ratio of immature (PCEs) and mature (NCEs) erythrocytes recorded from a total of 200 erythrocytes under trinocular microscope (Nikon, Japan).

## 3. Results

### 3.1. Ames Test

Cytotoxicity was not observed in terms of dose-dependent reduction in the number of revertant colonies and significant inhibition of background lawn, compared to the solvent control in the presence and absence of S9 in range finding study. Biological significance of the results was considered first. There was no concentration related or reproducible increase in the number of revertant colonies in the employed test concentrations in any of the tester strains. No two- or threefold increases in the means of the revertant counts was observed in the test concentrations in all tester strains with and without S9. Positive controls exhibited a significant multifold increase in revertant counts (*P* < 0.05, Dunnett's test). Thus, the negative result indicated that under these experimental conditions Pea Protein was nonmutagenic in the Ames *Salmonella typhimurium* reverse mutation assay (Table 2).

### 3.2. *In Vitro* Chromosomal Aberration Test

A moderate sedimentation of test substance was observed on slides in 125, 250, and 500 *μ*g/mL. Heavy compound sedimentation was observed at 1000 *μ*g/mL on slides in range finding study. Considering the noncytotoxicity and sedimentation of test substance, doses 125, 250, and 500 *μ*g/mL were chosen for the main study. In all the three treatment regimes (3-hour, ±S9 and continuous, −S9), treatment of cultures with Pea Protein Isolate resulted in an acceptable (−25.53% to 11.66%) %MI inhibition (Table 4). The percentage aberrations of all Pea-Protein-Isolate-treated cultures were not significantly different from the concurrent solvent control cultures in consensus with the scientific judgement (Table 3). Positive controls exhibited a significant increase in the percentage aberrations (*P* < 0.05, Dunnett's test). It is therefore concluded that, under the conditions of the test, Pea Protein Isolate did not induce genotoxic response in human lymphocytes when tested up to concentrations inducing acceptable levels of cytotoxicity:

(1)   %  Structural  aberrations   =Total  aberrations  from  duplicate  cultures(excluding  gaps)Total  no.  of  metaphases  scored  ×100.

### 3.3. In Vivo Micronucleus Assay

No mortality was observed in any of the groups. In the preliminary test there was a mild dose-dependent increase in the PCE : NCE ratio observed in females (PCE : NCE ratio of >1) without disturbance in cellularity, and a ratio of >1 was observed in males at 800 mg/kg b.w., exhibiting a similar trend to that of the concurrent vehicle control. In the limit test no evident increase in the frequencies of MN-PCE was observed in the dose group compared to that of the concurrent vehicle control groups in all time points of sacrifice (Table 5). However, an evident increase in the MN-PCE (>2 fold) was observed in the positive control group over the vehicle control and dose groups, thus validating the sensitivity of the assay (*P* < 0.05, Dunnett's test). From the previous results, giving credence to the scientific judgement, it was concluded that Pea Protein Isolate was nongenotoxic in single- and two-day treatments under the test conditions employed.

## 4. Discussion

In this paper, the possible toxic effects of the Pea Protein Isolate NUTRALYS were evaluated in first-barrier trials. Unifying all the information, the data suggest that the Pea Protein does not induce toxic effects, which could represent the safe implementation for nutritional supplementation of this product. Many nutritional supplement ingredients like Avemar pulvis, comprising fermented wheat germ, were tested for safety evaluation by acute, subacute, subchronic, and genetic toxicity studies and found to have no adverse effects at exposures far in excess of those that are expected to result from their intended use [6]. In its 2004–2009 Strategic Plan NIH's Office of Dietary Supplements looks for research assessing the effects of dietary supplements on biomarkers associated with chronic diseases, optimal health, and improved performance [7]. For this purpose genotoxic evaluation has been carried out to propose the genotoxic risk to the end user. The strategic recommendation of short-term genotoxic tests to assess the toxicity of the food ingredients [8] follows Ames bacterial reverse mutation test an *in vitro* chromosomal aberration test followed by an *in vivo* micronucleus test. This strategy has been implemented and the safety data was proposed for this food supplement. All three tests in the battery elucidated the non genotoxic nature of NUTRALYS Pea Protein Isolate and hence are recommended to be used as a dietary supplement for human, for which the humans equivalent dose was extrapolated from mice oral dose used in the micronucleus assay using the calculation from FDA guidance document—“Estimating the Maximum Safe Starting Dose in Initial Clinical Trials for Therapeutics in Adult Healthy Volunteers.”

Human equivalent dose in mg/kg b.w. = Mice dose in mg/kg b.w./12.3 = 2000 mg/kg b.w./12.3 = 162.6 mg/kg b.w.

## 5. Conclusion 

Finally NUTRALYS Pea Protein Isolate is considered nonmutagenic and nongenotoxic at the conditions employed in Ames test, *in vitro* chromosomal aberration test, and *in vivo* micronucleus test and suits a toxicologically safe protein supplement.

## Figures and Tables

**Table 1:** Positive mutagens for *Salmonella typhimurium* strains.

Method	Mutagen	No. of CAS	Solvent	Conc. per plate (*μ*g)	Responding *Salmonella* strains
Nonactivation (−S9) (Direct acting mutagens)	Mitomycin C (Sigma, USA)	50-07-7	Water	0.5	TA102
	Sodium azide (Sigma, USA)	26628-22-8	Water	1	TA1535, TA100
	2-Nitrofluorene (Sigma, USA)	607-57-8	DMSO	10	TA98
	9-Aminoacridine (Sigma, USA)	90-45-9	DMSO	50	TA1537
Activation (+S9)	Benzo(a)pyrene (Sigma, USA)	50-32-8	DMSO	10	All strains

**Table 2:** Summary of average histidine revertants and induction ratios.

Metabolic activation	Test concentrations (*μ*g/plate)	TA1535		TA1537		TA98		TA100		TA102	
		AHR ± SD	Induction ratio	AHR ± SD	Induction ratio	AHR ± SD	Induction ratio	AHR ± SD	Induction ratio	AHR ± SD	Induction ratio
Without S9	^ #^Solvent control	37.33 ± 2.08	—	9.00 ± 4.58	—	37.00 ± 4.36	—	200.00 ± 7.81	—	352.00 ± 11.27	—
	312.5	39.67 ± 7.02	1.06	10.33 ± 2.89	1.15	38.67 ± 7.09	1.05	214.00 ± 6.24	1.07	373.67 ± 15.63	1.06
	625	38.33 ± 7.37	1.03	7.33 ± 4.04	0.81	46.67 ± 9.45	1.26	203.00 ± 12.17	1.02	349.33 ± 18.82	0.99
	1250	31.00 ± 6.08	0.83	11.33 ± 2.89	1.26	36.67 ± 9.24	0.99	214.67 ± 4.04	1.07	324.33 ± 9.02	0.92
	2500	34.33 ± 4.62	0.92	12.00 ± 3.00	1.33	39.33 ± 7.77	1.06	209.33 ± 9.45	1.05	240.33 ± 5.51	0.68
	5000	24.33 ± 4.04	0.65	8.00 ± 3.61	0.89	29.67 ± 6.11	0.80	193.67 ± 13.05	0.97	242.00 ± 6.00	0.69
	Positive Controls	418.33 ± 21.22	—	121.00 ± 30.51	—	311.33 ± 2.89	—	1037.00 ± 53.36	—	1639.67 ± 44.64	—

With S9	^ #^Solvent control	37.33 ± 7.23	—	7.33 ± 2.08	—	36.67 ± 7.23	—	187.33 ± 8.02	—	386.67 ± 20.50	—
	312.5	31.33 ± 4.16	0.84	12.33 ± 3.06	1.68	29.00 ± 4.58	0.79	182.33 ± 12.50	0.97	381.67 ± 16.26	0.99
	625	28.33 ± 1.53	0.76	11.67 ± 5.03	1.59	26.67 ± 5.03	0.73	209.67 ± 2.31	1.12	383.33 ± 8.62	0.99
	1250	38.33 ± 2.08	1.03	9.67 ± 3.06	1.32	36.33 ± 10.07	0.99	191.00 ± 12.29	1.02	359.00 ± 3.46	0.93
	2500	25.33 ± 4.16	0.68	7.00 ± 1.73	0.95	48.33 ± 6.66	1.32	145.33 ± 15.89	0.78	377.33 ± 11.59	0.98
	5000	31.67 ± 10.26	0.85	7.67 ± 0.58	1.05	32.00 ± 4.00	0.87	136.33 ± 8.62	0.73	365.33 ± 10.41	0.94
	Benzo(a)pyrene (10 *µ*g /plate)	517.67 ± 64.63	—	76.67 ± 5.51	—	584.00 ± 36.37	—	1516.67 ± 13.58	—	1397.33 ± 421.17	—

AHR: Average histidine revertants, S.D.: standard deviation; induction ratio: mean number of revertants in treated/mean number of revertants in the control; ^#^sterile millipore water 100 *μ*L/plate.

**Table 3:** Summary of* in vitro *chromosomal aberrations in human lymphocytes.

Concentration (*μ*g/mL)	Total no. of metaphases scored	3 h with S9 (10%)		3 h without S9		Continuous without S9	
		% Numerical aberrations	% Structural aberrations (exclusive to gaps)*	% Numerical aberrations	% Structural aberrations (exclusive to gaps)*	% Numerical aberrations	% Structural aberrations (exclusive to gaps)*
Solvent control (sterile millipore water 50 μL/5 mL culture)	200	0	2.0	0	5.0	0	2.5
125	200	0	1.5	0	2.5	0	2.5
250	200	0	1.0	0	3.0	0	2.5
500	200	0	2.5	0	5.0	0	2.5
Positive control^#^	50	0	44.0	0	96.0	0	70.0

^#^20 μg/mL cyclophosphamide (with S9); 0.8 *µ*g/mL mitomycin C (without S9).

**Table 4:** Percentage Mitotic Index.

	% Mitotic index (MI)					
Concentration* (*µ*g/mL)	3h with S9 (10%)		3h without S9		Continuous without S9	
	%MI	% MI inhibition**	%MI	% MI inhibition**	%MI	% MI inhibition**
Solvent control (sterile millipore water 50 *μ*L/5 mL culture)	3.0	—	2.35	—	3.1	—
125^@^	2.65	11.66	2.4	−2.13	2.95	4.84
250^@^	2.9	3.33	2.95	−25.53	3.55	−14.52
500^@^	2.8	6.67	2.85	−21.28	2.85	8.06
Positive control^#^	2.6	13.33	1.7	27.66	1.65	46.77

*Mean value of duplicate cultures.

^#^20 *µ*g/mL cyclophosphamide (with S9); 0.8 *μ*g/mL mitomycin C (without S9).

^@^Moderate sedimentation of test substance on slides.

**% MI inhibition = ((% mean MI of solvent control − % mean MI of test concentration)/mean MI of solvent control) × 100.

**Table 5:** Summary of micronucleus assay.

Group/dose	Treatment/sacrifice	Total PCE scored		Total NCE scored		PCE : NCE		Total MN-PCE scored		%MN-PCE Mean ± SD	
	Time point	M	F	M	F	M	F	M	F	M	F
G1 millipore water	Single/24 h Single/48 h Two day/24 h*	563 569 528	501 511 490	437 431 472	499 489 510	1.30 1.34 1.14	1.01 1.05 0.98	10 11 16	12 10 13	0.10 ± 0.08 0.11 ± 0.05 0.16 ± 0.04	0.12 ± 0.08 0.10 ± 0.04 0.13 ± 0.08

G2 2000 mg/kg b.w.	Single/24 h Single/48 h Two day/24 h*	541 563 520	539 555 483	459 437 480	461 445 517	1.18 1.31 1.09	1.18 1.26 0.94	10 15 15	12 14 15	0.10 ± 0.05 0.15 ± 0.08 0.15 ± 0.07	0.12 ± 0.06 0.14 ± 0.04 0.15 ± 0.04

G3 40 mg/kg b.w. CYP	Single/24 h	506	528	494	472	1.04	1.13	92	80	0.92 ± 0.18	0.80 ± 0.22

24 h*: Following last administration; M: male; F: female; CYP: cyclophosphamide (monohydrate); PCE: polychromatic erythrocyte; NCE: normochromatic erythrocyte; MN-PCE: micronucleated polychromatic erythrocyte.
